# A narrative action on the battle against hunger using mushroom, peanut, and soybean-based wastes

**DOI:** 10.3389/fpubh.2023.1175509

**Published:** 2023-05-11

**Authors:** Nurul Aqilah Mohd Zaini, Nur Asyiqin Zahia Azizan, Muhamad Hafiz Abd Rahim, Adi Ainurzaman Jamaludin, António Raposo, Siva Raseetha, Renata Puppin Zandonadi, Mona N. BinMowyna, Dele Raheem, Linda Heejung Lho, Heesup Han, Wan Abd Al Qadr Imad Wan-Mohtar

**Affiliations:** ^1^Department of Food Sciences, Faculty of Science and Technology, Universiti Kebangsaan Malaysia, Bangi, Selangor, Malaysia; ^2^Functional Omics and Bioprocess Development Laboratory, Institute of Biological Sciences, Faculty of Science, Universiti Malaya, Kuala Lumpur, Malaysia; ^3^Faculty of Food Science and Technology, Universiti Putra Malaysia, Serdang, Selangor, Malaysia; ^4^Environmental Management Programme, Institute of Biological Sciences, Faculty of Science, Universiti Malaya, Kuala Lumpur, Malaysia; ^5^CBIOS (Research Center for Biosciences and Health Technologies), Universidade Lusófona de Humanidades e Tecnologias, Lisboa, Portugal; ^6^Faculty of Applied Sciences, Universiti Teknologi MARA, Shah Alam, Selangor, Malaysia; ^7^Department of Nutrition, Faculty of Health Sciences, University of Brasília, Brasília, Brazil; ^8^College of Applied Medical Sciences, Shaqra University, Shaqra, Saudi Arabia; ^9^Northern Institute for Environmental and Minority Law (NIEM), Arctic Centre, University of Lapland, Rovaniemi, Finland; ^10^College of Business, Division of Tourism and Hotel Management, Cheongju University, Cheongju-si, Chungcheongbuk-do, Republic of Korea; ^11^College of Hospitality and Tourism Management, Sejong University, Seoul, Republic of Korea

**Keywords:** circular economy, future food, mushroom, peanut, soybean, zero food hunger

## Abstract

Numerous generations have been affected by hunger, which still affects hundreds of millions of people worldwide. The hunger crisis is worsening although many efforts have been made to minimize it. Besides that, food waste is one of the critical problems faced by most countries worldwide. It has disrupted the food chain system due to inefficient waste management, while negatively impacting the environment. The majority of the waste is from the food production process, resulting in a net zero production for food manufacturers while also harnessing its potential. Most food production wastes are high in nutritional and functional values, yet most of them end up as low-cost animal feed and plant fertilizers. This review identified key emerging wastes from the production line of mushroom, peanut, and soybean (MPS). These wastes (MPS) provide a new source for food conversion due to their high nutritional content, which contributes to a circular economy in the post-pandemic era and ensures food security. In order to achieve carbon neutrality and effective waste management for the production of alternative foods, biotechnological processes such as digestive, fermentative, and enzymatic conversions are essential. The article provides a narrative action on the critical potential application and challenges of MPS as future foods in the battle against hunger.

## 1. Introduction

International organizations such as the Food and Agriculture Organization (FAO), World Health Organization (WHO), and World Food Program (WFP) describe hunger in multiple different ways. These include chronic undernourishment, a shortage of food supply, food insecurity, reduced food intake accompanied by physical symptoms brought on by hunger, and continual anxiety about where and when their next meal will be (1). In 2021, between 702 and 828 million people experienced hunger (2) and more than 1 billion individuals worldwide suffer from hunger and malnutrition as well as food insecurity, with a large portion of this population living in Sub-Saharan Africa and South Asia. As a result of undernutrition and child mortality, the global hunger index has been rising (3).

According to FAO (2), world hunger increased even more in 2021, contrary to expectations that the outbreak of COVID-19 would be over and food security would start to improve. By 2021, approximately 30 percent of the world’s population, or about 2.3 billion people, were either moderately or severely food insecure. The world population was expected to reach 8 billion on November 15, 2022, will approach 9 billion around 2037, and is predicted to hit 10 billion around 2058 (4), which would result in 50, 60, and 55% rises in the needs for water, energy, and food, respectively (5, 6). Further implications and opportunities follow from this prediction. High production wastage is one of them. The World Bank predicts that, from the anticipated value of 2.01 billion tonnes per year in 2016, worldwide waste will increase by over 70% by the year 2050 (7).

The circular economy’s fundamental philosophy is to actively advance and recognize each step of production in order to reuse the wastes generated by industrial activities (8). Agriculture has been seen as a pertinent field for the circular economy’s application due to its concerns with environmental sustainability, significant waste output, and nutrient flow limitations (9). Based on Supplementary Figure S1, using the concept of circular economy, agricultural food wastes from mushrooms, peanuts, and soybean are no longer viewed as worthless entries but rather as precious resources to produce future food. The ever-increasing human demand for protein-rich food and the inadequacies of existing technologies have necessitated the search for low-cost options for producing alternative protein-rich meals (10). In Nigeria, where people look to mushroom and their cultivation to combat poverty, hunger, and malnutrition, mushrooms are a fantastic illustration of how food security may be achieved since they give a significant level of fiber and protein (11). The beneficial monounsaturated fat in peanuts may regulate hunger and satiety which enables a person to feel full after ingestion (12). Other than mushroom and peanut, soybean-based food is a crucial tool for addressing hunger and malnutrition issues. With the least amount of N fertilizer input possible, farmers grow soybeans to increase yields, family demand, and net profits, thus raising their standard of living and ensuring their food security (13).

The production of mushrooms has increased globally as well, stemming from approximately 53 million tonnes of mushroom waste (1 kg of mushrooms requires 5 kg of mushroom media substrate) (14). Around 12.74 million tonnes of mushrooms are consumed worldwide at the moment and statistics show that by 2026, the value of the world mushroom market is predicted to reach 20.84 million tons (15, 16). Besides mushroom, the amount of peanuts produced worldwide in 2019 was 45.06 million tons (17). In 2020, up to 54 million tonnes of peanuts were cultivated, with the majority coming from China (34%), an increase of 8% from the preceding year (18). From 2014 through 2019, soybean-producing nations’ year-end stocks ranged from 34 to 52 million tonnes. The countries that produce soybeans are anticipated to complete the production year with roughly 47 million tonnes of inventories, with this trend forecast to continue into 2020 (19). In 2030, a rise in global soybean production of 371.3 million metric tonnes is predicted (13).

## 2. Mushroom

From 14,000 known species worldwide, an estimated 1.5 million fungi produce fruiting bodies large enough to be classified as mushrooms (20). There are currently about 2,000 edible varieties of mushrooms spread throughout the world, making mushrooms a fungus with high nutritional values (21). Mushrooms are low in fats, but possess an important protein content, achieving up to 35% (dry weight), and contain nine essential amino acids (histidine, isoleucine, leucine, lysine, methionine, phenylalanine, threonine, tryptophan, and valine) that comprises about 20 mg/g on fresh weight basis (22). Different varieties of mushrooms have different colors, forms, textures, and behaviors (23). Mushrooms are primarily composed of moisture (85–95%), followed by carbohydrates (37–70%), protein (15–34.7%), fat (10%), minerals (6–10.9%), and nucleic acids (3–8%). Additionally, it includes a lot of vitamins, including riboflavin (6.7–9.0 mg/100 g), thiamine (1.4–2.2 mg/100 g), niacin (60–73.3 mg/100 g), biotin (0.6–0.7 mg/100 g), pantothenic acid (21.1–33.3 mg/100 g), ascorbic acid (92–144 mg/100 g), and folic acid (1.2–1.4 mg/100 g) in dry basis (24). Due to their biological effects, mushroom extracts exhibited antioxidant, antibacterial, anti-inflammatory, anticancer, anti-obesity, and immunomodulatory properties (25). The phytochemical components of mushrooms are, therefore, of great interest to nutritionists, and consumers as they have positive health effects on humans and lower the risk of disease (26).

There are numerous substrates for mushrooms, and the mushroom industry is one of the largest producers in the world. However, there are concerns with their storage and disposal. One of the most popular edible mushroom species grown industrially is *Pleurotus* spp., which represents more than 25% of the mushrooms produced globally (27). High waste mushroom substrate (WMS) results from increased mushroom production. Extracellular lignocellulosic enzymes, organic molecules (including proteins, carbohydrates, and lipids), fungal mycelium, and inorganic compounds, including ammonium nitrate, are all present in high concentrations in WMS (28). Due to the increase in mushroom cultivation, finding a new application for spent mushroom substrate (SMS) is necessary because there is now an excess of SMS output, which poses a waste management issue. Supplementary Figure S2 shows an example of a commercial yellow oyster mushroom with three biomasses: fruiting body-base (FBB), fruiting body, and SMS.

The fruiting body of a mushroom is defined as all the fungi’s propagule-producing and -distributing structures that arise from their fungal mycelia originating from hyphal branches (29). The species, developmental stage, and environmental factors of mushrooms all have a significant influence on their nutritional value of mushrooms. For instance, according to Isaac et al. (30), *Pleurotus ostreatus*’s protein contents varied noticeably, with the cap having 34.19 g/100 g, the stalk having 20.96 g/100 g, and the cap with a stalk having 30.48 g/100 g. In a similar study, there is high content of dietary fibers and polysaccharides, especially in the cap and stalk of *Pleurotus ostreatus* (31).

FBB is frequently disposed of as waste, while only the stem and cap are harvested for industrial use (32). Many discarded FBB was converted into FBB flour and utilized to create food products such as cookies, steamed bun (32), and mushroom-chicken patty (33). Both studies have proven FBB flour as a functional source of antioxidants and are accepted by consumers.

SMS stands for mushroom production biomass waste that contains a variety of components, such as extracellular enzymes and fungus mycelia (34). The annual volume of SMS produced by the mushroom industry presents a substantial disposal difficulty. In the past three decades, there has been an upsurge in concerns over the disposal of SMS. Furthermore, the practice of growing mushrooms has increased, and every year there are around 5 million tonnes of solid waste created (34). As a result of its full integration into the economic cycle of resource reutilisation for energy production, SMS is now a valuable resource for biogas production (35). Besides that, bioethanol is produced using the SMS of *Ganoderma lucidum* (10–50% dry weight; DW) containing a high level of polysaccharides such as fructose, mannose, rhamnose, glucose, and xylose to meet the biofuel demand (36).

### 2.1. Mushroom as a functional food and nutraceutical ingredient

Mushroom is increasingly regarded as a future food due to their functional and nutraceutical properties. Functional food is described as food that, in addition to providing nutritional benefits, favorably influences one or more biological functions. It is not a supplement or tablet but rather a natural component of a person’s diet (37). On another note, nutraceuticals can be defined as a material that is classified as food or a component of a meal that offers medical or well-being purposes, such as the prevention and treatment of illness (38). Given its low-calorie content, pleasant flavor, and reputed beneficial biological activities, the mushrooms’ extracts and the mushroom have been utilized in traditional medicine and food since the ancient era (39). It is typically taken as dietary supplements in the form of capsules to control cardiovascular disease, diabetes, cancer, and hypertension; boost the immune system; possess antiviral and antibacterial capabilities; and engage in wound healing activities (40). Supplementary Table S1 shows the recent applications and products of various mushroom species.

Both the antimicrobial and antibacterial properties of mushrooms have been shown in several studies. Based on a study by Hassan et al. (41), the strongest inhibition was seen in the *Ganoderma lucidum* and *Laetiphorus sulphureus* extracts, with a minimum inhibitory concentration (MIC) of 0.1 mg/mL. A significant antibiotic effect was also demonstrated by water extracts of the following plants: *G. applanatum*, *Trametes versicolor*, *Polyporus squamosus*, *Lentinellus subaustralis*, *Laetiporus sulphureus*, *Ganoderma lucidum*, against all tested bacterial strains (*Bacillus subtilis, Micrococcus luteus, Pseudomonas aeruginosa* reference strains PAO1 and PA14, *Staphylococcus epidermidis and P. fluorescens*). The antibacterial activity of *Agaricus bisporus* S-II was effective against a variety of infections (i.e., *P. aeruginosa* MTCC741, *B. cereus* MTCC9786, and *S. aureus* MTCC740) (42). Besides that, *B. subtilis* and *Escherichia coli*’s growth was also inhibited by extracts of fresh or dried fruiting bodies of *Pleurotus ostreatus*’s mycelium (43).

### 2.2. Mushroom in landless food concept

As mushroom cultivation incorporates the reuse of agricultural and agro-industrial waste in the manufacture of food with a high nutritional value, it stands out as an environmentally friendly technique (44, 45). In recent years, the use of mushroom cultivation techniques to reuse or dispose of solid organic wastes has become increasingly important. According to Grimm et al. (46), mushroom cultivation has a significant role in agricultural systems because it can be used to recycle biomass and byproducts from crops and, particularly, animal husbandry. Integration of mushroom cultivation across these two systems could lead to increased output and more efficient use of resources.

In order to help accelerate cultivation and enhance productivity, submerged liquid fermentation of *Ganoderma lucidum* has been proven to become the fast cultivation method; approximately 10 days (47) compared to traditional mushroom cultivation, which requires at least 6 months to grow (48). Solid-state fermentation (SSF) is a process in which, in the absence (95% onwards) of free water, solid particles with a continuous gaseous phase between them act as either a substrate or an immobile solid support for the development of microorganisms (49). The active ingredient’s functioning is severely limited due to the prolonged culture period and the inconsistent fruiting body quality of *Ganoderma lucidum* in the typical environment (50). Given its short production cycle, controlled procedure, high output, consistent product quality, and low cost, liquid-submerged fermentation has emerged as a novel and significant technique for extracting the bioactive components of *Ganoderma lucidum* (51).

Mushroom has been widely applied as additive and replacer in various food products (32, 33, 52, 53). Despite adopting multiple approaches that alter the structure of mushrooms or increase their ability to hold water, there are still various issues involving color and water absorption compared to meat (54). As a result of water absorption and how water attaches to proteins and their fibers, meat has a distinctly juicy quality. In addition, meat products have texture and palatability concerns as well as a crimson, reddish, or pink coloring that is difficult to obtain without using colorants (55).

## 3. Peanut

Peanut is categorized under the Fabaceae family, including peas, legumes, and beans. It is consumed worldwide due to their rich nutritional properties and are a staple ingredient in many dishes. Perhaps, its most common utilization as a snack is when prepared directly after harvesting. It is highly nutritious, whereby up to half its weight comprises edible oil (long-chain monounsaturated fatty acids). In terms of other nutrients, up to 24% of its weight is in the form of easily digestible proteins, 35% of total essential minerals, and almost half of 13 essential vitamins can be obtained from this “longevity fruit” (56). Nevertheless, there can be certain risks associated with peanut consumption, especially due to the presence of *cupin* and *prolamin* protein superfamily that may cause allergenicity (57), and food safety due to improper storage of peanuts that may cause aflatoxin poisoning due to *Aspergillus* or *Penicillium* fungal growth (58).

According to recent data, most peanuts are processed for their oil, except in the United States, where 80% of its peanut is turned into peanut butter (59). Due to the protein content, peanuts can also be used to produce flour and protein concentrates, which are suitable for gluten-free food application. Peanut oil is a type of vegetable oil that is mild in taste, has a high smoke point, and low rancidity. Together, these characteristics lead to the significant use of peanuts, which is currently just behind that of soybean in terms of legume production. In 2020, up to 54 million tonnes of peanuts were cultivated, with the majority coming from China (34%), an increase of 8% from the preceding year (18).

Apart from being nutritious with a high yield, the cultivation of peanuts is simple, coupled with the ability of the crop to replenish the soil nutrients through nitrogen-fixing bacteria. Currently, as illustrated in Supplementary Figure S3, there are plenty of non-food applications of peanut waste. The simplest way of utilizing these wastes is by producing biofertilizers (aerobic/composting or anaerobic digestions that produce biogas as a by-product), or by feeding it to the animals due to the high protein contents, especially in the spent (60). Therefore, it is desirable to utilize every bit and piece of peanut waste during processing into high-value food products to achieve total economic circularity.

### 3.1. Peanut shell and waste applications

During the processing of peanut-related products, the main waste being produced is its shell (Supplementary Figure S3), which accounted for up to 20% of the dried peanut pod by weight (60). During peanut oil extraction, the leftover (known as peanut cake) can reach up to 3.26 million tons per year, considering 45% oil yield in the peanut (61). The pink-red layer that covers the peanut kernels, known as peanut skin, is still regarded as a substantial by-product of the peanut processing industry, with 0.94 million tonnes produced per year. Conversion of peanut skin into food-related applications poses a challenge in terms of cost (extraction and enzyme pre-treatments), safety (presence of aflatoxin, allergens, and heavy metals), and quality (high amount of antinutrients and fibers).

Peanut shells are primarily composed of lignocellulosic materials with trace components of proteins, fats, sugar, minerals, and several bioactive compounds such as polyphenols, flavonoids, luteolin, and carotene (62). The fibers contain a rich amount of cellulose, hemicellulose, pectin, and lignin, with good water-holding capacity and nutritional and physicochemical properties (63). In an investigation by Adhikari et al. (62), it was shown that peanut shells from various Korean cultivars contain a high level of antioxidants ranging from 428.1–739.8 μg gallic acid equivalents/g for polyphenols, 142.6–568.0 μg quercetin equivalents/g for flavonoids, and 5.76–34.56 mg/g of amino acid. Further evaluation revealed a total of 29 amino acids in the shell, which contributed to different human physiological functions, ranging from muscle movement to signaling regulations (62). The shell has been identified to contain various important bioactive compounds for pharmaceutical industries, isolated from bio-oils (64), various extracts (65), and antioxidants from methanolic extract (66). These components are isolated using various chromatographic methods of LC–LC, LC-TOF-MS, and DPPH-HPLC-DAD-TOF/MS. To achieve total circularity, the residual shell, after extraction, could be utilized in a biorefinery system as a source of fibers to fortify food products (61).

Among the three major wastes of peanuts, its shell is likely the least suitable candidate for valorisation in food-related applications. Peanut shell is the main remainder of peanut processing, which accounts for up to 20% of the dried peanut pod or 9.2 million tonnes annually (61). Unfortunately, due to its high cellulose, hemicellulose, and lignin, this waste is difficult to utilize and slow to biodegrade or digest (60). A common method to dispose of this waste is either being burnt off or buried, which can lead to wastage and environmental concerns. Therefore, most applications involved non-food components, such as pulp, composite and building materials, biosorbents, wastewater treatment, nanomaterials, and the biofuel industry (60). Supplementary Table S2 demonstrates the possible applications of peanut shells in the food industry. Most of the current research focuses on the extraction methods of the shell and its corresponding bioactive compounds, with limited information *in vitro*, *in vivo,* and industrial food applications. As a result, it showed that the study into the use of peanut shells is still in its early stages, perhaps as a result of the difficulty in extracting the different parts of peanut shells.

### 3.2. Peanut meal and waste applications

Peanut meal is the peanut spent or cake obtained after producing peanut oil. To improve its utilization, cold press or non-thermal processing of peanut oil is desirable to preserve the nutritional qualities of the residue (63). This waste is likely to have the most nutrient composition compared to the other wastes, especially protein at about 50% and carbohydrate at about 32% of the dry basis. Therefore, peanut cake is often utilized as animal feed, which has been demonstrated to increase meat safety and quality (67) and their associated products, such as milk, to produce cheese (68). However, converting peanut cake into high-value applications is desirable due to its rich nutritional profiles. Some of the applications of peanut meal in high-value food applications are demonstrated in Supplementary Table S3.

The general carbohydrate contents of peanut meal are low and comprise monosaccharides (0.31%) in the form of fructose (0.12%) and glucose (0.19%). The oligosaccharides comprise11.09% of carbohydrate content in the form of sucrose (9.83%), maltose (0.74%), lactose (0.42%) and stachyose (0.1%), and polysaccharides (21.05%) in the form of starch (8.01%), crude fiber (3.64%) and heteropolysaccharides (9.4%) (63). The polysaccharides can serve as a functional addition to food formulations, as they have many different physiological effects *in vitro* and *in vivo* (63). If the purpose of the peanut meal is to extract the polysaccharides, the proteins are likely to be removed first to assist in the extraction.

Peanut meal is rich in proteins and several essential amino acids. In a characterization made by Batal et al. (69), five different variants of peanut meal demonstrated an average of 2,664 kcal/kg of nitrogen-corrected metabolizable energy, with 45.6% of crude protein (69). Compared to soybean meal, it is low in lysine (1.54%), but is rich in arginine (5.04%), with an excellent digestibility index of over 80% in all 17 amino acids tested. By using appropriate processing methods such as fermentation or enzymatic hydrolysis, high values protein hydrolysates such as functional oligopeptides could be produced for food-based applications (63). These peptides could confer multiple health or pharmaceutical benefits, ranging from antioxidant compounds, enzyme inhibitors, antihypertensive, antimicrobials, and others (70). However, the two major protein globulin fractions of peanut – *arachin* and *conarachin* from *ara* family proteins, can be an allergen to sensitive individuals (71). Interestingly, these two fractions contributed to the fibrous meat-like properties, and thus can potentially be converted into meat alternatives (72). This condition is similar to gluten in bread, whereby allergenic proteins are the major contributor to the texture of the food product. Therefore, as long as the individuals are not immunologically susceptible to the *ara* protein family of peanuts, it can be an excellent source of protein with desirable organoleptic properties. Further proteolytic enzymatic pre-treatment or physical processing, e.g., extrusion or heat treatment may be considered to break the allergens if the product is to be taken by an allergic person (73).

As the peanut oil is extracted, some fat-related micronutrients such as Vitamin E are likely to be reduced. However, the content of other water-soluble vitamins, such as vitamin B (peanut is rich in folic acid), and minerals such as iron, zinc, and calcium (74) might be retained if non-thermal processing is employed. Due to the presence of antinutritive compounds in peanuts, fermentation to degrade these compounds is needed to improve the bioavailability of minerals for absorption (74), while modulating the microbiota population through the gut-brain axis (75). Nevertheless, there is limited information on the micro-nutrients (vitamins and minerals) in the peanut cake waste, compared to the information on polyphenols which are, sometimes, known as the “seventh nutrient class.” Like peanut skin, peanut meal may possess a high amount of A-type procyanidins, resveratrol, and other phenolics (76).

### 3.3. Peanut skin and waste applications

Compared to the other parts of the peanut, the skin is perhaps the most useful part of the waste that can be used for food applications, but it only represents around 3% of total peanut weight (77). The skin is often removed due to the potential off-color development, undesirable (bitter or astringent) taste, and possible contamination by aflatoxin-producing fungi. The skin is rich in polyphenols, specifically, proanthocyanidins, which makes it useful for various food applications involving food preservation to retard oxidation and microbial growth (77), and as functional foods such as antidiabetic, cardioprotective, and anticancer (Supplementary Figure S3). Unlike the shell or the cake, the skin is low in macronutrients but with a high amount of tannins, unsuitable for animal feed (77, 78). Although tannins chelate the minerals and hence reduce their bioavailability, they can be an excellent biosorbent to remove heavy metals (65) or in dye production (79). The presence of many phenolic and flavonoid compounds encourages researchers in the area of new compound identifications and optimisation of extraction parameters, as demonstrated in Supplementary Figure S4 (65).

Several food applications take advantage of polyphenols’ superior antioxidant content to increase the shelf-life of the food product. In addition to protecting human health by scavenging free radicals, antioxidants can lessen protein and lipid oxidation by stopping the chain reactions that could otherwise damage macromolecules. While roasting peanuts, leaving the skin intact might help reduce the accumulation of polycyclic aromatic hydrocarbons due to the antioxidative effect of the skin (80). In a recent review by Lorenzo et al. (77), the antioxidative capacity of peanut skin extract (PSE) was highlighted to enhance the shelf-life of meat goods by the action of phenolics in arresting the free radical’s propagation, depigmentation, and microbial growth. Furthermore, Christman et al. (81) have demonstrated the potential of antioxidants of the PSE in chili lime and honey-roasted coatings of peanuts without influencing their sensorial acceptance (81). Similar sensory conclusions were also demonstrated elsewhere (82, 83). However, high usage of PSE is undesirable due to the tendency of proanthocyanin to induce bitter and astringency flavor (82).

Similarly, the therapeutic effect of PSE has been attributed to its polyphenol contents, especially procyanidins and resveratrol. These compounds can inhibit the multiplication of cancerous cells via several mechanisms, such as induction of cell death, regulation of signal transduction, and inactivation of histone deacetylase. These effects were shown in different cancer cell models, such as prostate cancer (84), melanoma (85), and carcinoma cell line (86). The polyphenols from PSE were shown to have anti-allergic effects through Mitogen-Activated Protein Kinase (MAPK) signaling pathway (87) and antidiabetic properties through improvement in lipid profiles (87), inflammation, and gut microbiota (88). In more interesting applications, PSE was shown to ameliorate brain conditions through antioxidative and signal transduction (89), as skin lightening (90), anti-anxiety and analgesic agent (78), and as an antiviral agent for influenza virus (91).

The main challenge in utilizing peanut skin is the optimal procedure to isolate the proanthocyanidins, which requires multiple steps. Additionally, the antimicrobial property of peanut skin is not wide-ranging, as only several food pathogens were shown to be selectively inhibited by the extract. For example, peanut skin was demonstrated to have weak inhibition against *Salmonella typhimurium* and *Listeria monocytogenes* compared to grape extract, which was attributed to the presence of A-type procyanidins instead of more potent B-type procyanidins in grape seed (92). While in another study, the peanut skin extract stimulated the mycotoxin fumonisin B1 production in *Fusarium verticillioides* (93), which is undesirable. The extract was also ineffective at the lower concentration on yeast growth, which makes it impractical for food application (94). Additionally, the extract also inhibited commensal (natural and good probiotic bacteria) lactic acid bacteria *Lactobacillus rhamnasus*, *L. plantarum,* and *L. casei*, especially if PSE is added as an additive in fermented food (92).

It is worth mentioning that peanut is highly susceptible to aflatoxin and salmonella contamination. The environmental condition of peanuts is vital to combat aflatoxin contamination. Aflatoxin produced by *Aspergillus parasiticus* and *Aspergillus flavus* can grow during pre-harvest and poor storage, thus, posing the risk of fatally chronic illness and child mortality (95). Due to its widespread usage as food ingredient, peanut increased the severity and financial impact of *salmonellosis* outbreaks (for example, a 2009 outbreak involving peanut butter resulted in over $1 billion in losses owing to product recalls and nine deaths) (96).

## 4. Soybean

Skyrocketed uilisation of plant-based proteins in the food industry especially from soy has been observed due to its high protein content (35–45%) and the quality of protein. In Asian countries, various foods are made of soy, including soybean oil soymilk, soy flour, soy sauce, and tofu. Protein from soy contains well-balanced essential amino acids and can be compared with other protein sources food such as eggs, milk, and meat. The amino acid composition of soybean is comparable with animal proteins, such as leucine, isoleucine, lysine, tryptophan, methionine, valine, phenylalanine, and threonine (97). Thus, soy-based protein products have been recognized as the primary replacement for animal foods in the vegan dietary guideline. Soybean has also been intensively used in the food industry because of its functional properties which can influence the end-product characteristics. As a result, soybean is regarded as one of the most significant plant-based proteins that are widely consumed and used as a crop around the world, particularly in Asia (98).

With the increased production and consumption of soybean, there has been a corresponding increase in organic waste generation. Apart from being used as compost and animal feeds, some manufacturers disposed of the generated waste in landfill (99). Since most agricultural wastes contain a high composition of nutrients, a serious environmental problem associated with odors and leachates due to pest infestation, microbial domination and harmful gaseous production are expected. Thus, the reutilisation of soybean waste is one of the approaches to be taken in safeguarding environmental sustainability. The commonly generated by-product or wastes from soy processing are okara (sometimes referred as soybean curd leftover), soybean meal, soy-whey, and soy hull. Supplementary Figure S5 shows the general processing flow involved in soybean processing with the generation of respective waste. While Supplementary Table S4 summarizes the potential direct application of soybean waste for the food industry.

Okara, a white-yellow fibrous residue, is one of the major agricultural wastes generated from processing soymilk, tofu, and their derivatives. It is the insoluble portion of the soybean. According to Choi et al. (99), around 250 kg of okara was generated from 1,000 L productions of soymilk. They projected that approximately 14 million tonnes of soybean waste are produced annually. Although okara is considered as a waste, it contains significant amount of crude protein (20.9–39.1%), crude fiber (12.2–61.3%), crude fat (4.9–21.5%), and ash (3.4–5.3%) in dry basis (100). For crude fiber composition, approximately 50% of the dried okara comes from cellulose and hemicellulose, which can be further hydrolyzed to glucose (11.9–15%), galactose (10.4–10.8%), arabinose (5.7–6.4%), xylose (2.7–5.1%) and mannose (1.3–1.5%). Besides, various types of minerals were also found in okara, such as sodium, potassium, magnesium, and calcium, as reported by Qin et al. (98).

However, the usage of okara is restricted due to its quick decomposition and putrefaction because of its high moisture content (70–80%) (101). Currently, okara is used as animal feed, dumped in landfill, or plant fertilizer even though it is renowned for its excellent functional properties. Due to its significant amount of protein and fibers, a lot of research has been conducted to reutilise okara to produce high-value products.

Soybean meal is one of the key by-products (80%) of the soy processing industry, specifically from soybean oil extraction. It is the most common protein supplement used for livestock, including ruminants and poultry worldwide, as it comprises a substantial quantity of crude protein (32–45%) and fat (20%) (100, 102). As soybean meal comprises a high amount of digestible protein, it is also being used as an alternative protein source for fish and shrimp. The composition of other nutrients, such as carbohydrates, is between 31.7 and 31.85%, while crude ash is in the range of 4.5–6.4% (103). In addition, soybean meal is rich in isoflavones, which are linked with antioxidant activity that reduce the formation of radicals. However, the nutritional content of soybean meal varies depending on where the beans were grown. For example, Ibáñez et al. (104), demonstrated that, soybean meals from Brazil contain more crude protein and less sucrose concentration.

In addition to simple sugars, proteins, oligosaccharides, soy isoflavones, and minerals, soy-whey is a by-product of the manufacturing of both soy and tofu protein isolate (105). According to Fei et al. (106), for every kilogram of soybean utilized to produce tofu, about 9 kg of tofu whey is generated. Soy-whey is frequently disposed off into sewage as an effluent during the process. However, it contains high organic compounds and water content that further require expensive wastewater treatment before being disposed off.

Soy hull is another product of the soy processing industry that represents around 5–8% of the whole soybean seed. Soy hull is an excellent supplemental feed for dairy cattle. The main composition of soy-hull is fiber comprised of cellulose (28.6–52.3%), hemicellulose (3.1–33.8%), and pectin (2.1–13.1%) (107). Thus, soy-hull is considered the main polysaccharides byproducts from the soy processing industry compared to soybean meal, okara, and soy-whey.

### 4.1. Prospective application in food industries, agricultural and energy

In general, there are two common approaches taken to reutilise okara in the food industry. Firstly, by directly utilizing okara as a functional ingredient or dietary supplement. Bedani et al. (108) study the effect of the incorporation of okara in soy yogurt fermented with *Lactobacillus acidophilus* La-5, *Bifidobacterium animalis* Bb-12, and *Streptococcus thermophilus*. They found that the firmness of yogurt was increased, and the sensory acceptability was not affected, suggesting that okara might help increase probiotic soy yogurt’s nutritional and functional properties. Okara was also incorporated in probiotic ice-cream as reported by Ibrahim et al. (109). The research revealed that okara supported the growth of *L. plantarum* and slowed down the melting rate of ice cream. A similar finding was reported, where okara was proven as a good source in modulating the gut microbiota in a high-fat diet in Wistar Hannover male rats, thus, showing potential prebiotic effects (110). Besides, okara boosted the fiber and protein content when incorporated into the production of gluten-free cookies (111). Okara has also been used to substitute wheat flour in butter cake processing. Results showed that 20% okara lead to higher oil and water absorption capacities, total phenolic content, fiber, and protein, and more acceptable sensory profiles than wheat flour (112). These researchers have demonstrated that the high nutritional value of okara can be employed in the food industry especially as a substitute for typical flour to raise the protein and dietary fiber content of food products that would provide an alternative to common food sources and consequently combat food hunger.

The second approach is by bioconversion of okara as raw material to produce valuable compounds. Normally, okara will first be converted to monomeric sugar such as glucose or galactose by enzymatic or chemical (alkaline or acid) hydrolysis, before being used as a substrate for fermentation. For example, bioethanol production of okara by *Saccharomyces cerevisiae* shows a higher ethanol conversion yield (96.2%) from okara’s sugars during fermentation (99). Erythritol was another compound that was successfully produced from okara through a two-stage solid state fermentation (SSF): (pre-fermentation with *Mucor flavus* and *in situ* erythritol fermentation with *Yarrowia lipolytica*) (113). Besides, okara was also used as a substrate for microalgae *Phaeodactylum tricornutum* production as recently reported by Kim et al. (114). The result indicated the increments in biomass production by two-folds, fucoxanthin by two-folds, and polyunsaturated fatty acids, particularly eicosapentaenoic acid (EPA), by approximately three-fold.

Soybean meal is commonly applied as animal feed due to its high protein content. However, research on the direct reutilisation of soybean meal in other areas is limited. There are few findings on the reutilisation of soybean meal in food applications, mainly biscuits. However, a combination with other approaches, such as enzymatic hydrolysis or fermentation, is needed before being accepted by the consumer (115, 116). In terms of the bioconversion approach, soybean meal shows potential to be used as a substrate for ethanol production replacing sugar cane, corn, or beets, due to its high carbohydrate content (117). Their study found that *Zymomonas mobilis* subsp. *mobilis* NRRL B-4286 and *Saccharomyces cerevisiae* NRRL Y-2233 was able to produce 4 g ethanol/100 g fresh soybean meal and 4.6 g ethanol/100 g fresh soybean meal, respectively, once being pre-treated with dilute-acid hydrolysis. On the other hand, soybean was also evaluated for its potential as an economical nitrogen source during lactic acid fermentation to substitute yeast extract (118). Although the result shows slightly lower production of lactic acid from soymeal (162.5 g/L) compared to yeast extract (180 g/L), the cost for nitrogen sources was estimated to be only 25% of that with yeast extract, suggesting by incorporating soybean meal as a low-cost nitrogen addition can help cut the cost of producing lactic acid.

Moreover, researchers have investigated several methods to valorize soy-whey. In general, there are two types of valorisation procedures: physical methods (which attempt to extract nutrients) and microbiological or enzymatic approaches (for biotransformation). Soy whey can be extracted using a variety of methods, including ultrafiltration, electrodialysis, and bipolar-membrane electro-acidification, because it contains a significant amount of nutrients. For instance, using ultrafiltration and various diameters of membranes with varied molecular weight cut-offs (MWCO), soy protein and oligosaccharides can be separated from soy-whey (105). Besides, soy isoflavones, a bioactive compound from soy, had been successfully recovered by Liu et al. (119) using foam fractionation followed by an acid hydrolysis method with 87.72% recovery yield. In biotransformation, the aim is to utilize soy-whey as a whole medium for producing valuable compounds such as ethanol and organic acid for food or non-food applications. For example, Zhu et al. (120) successfully produced soy-based probiotic drink rich in isoflavone aglycones utilizing soy-whey using *Lactobcillus rhamnosus* GG and *Lactobacillus paracasei.*

Researchers have recently begun to look at the potential of soy-hull as sugar-rich fermentation feedstock for bioethanol, enzyme, and organic acid production as an alternative to conventional starchy substrate. For example, Mielenz et al. (121) have successfully produced ethanol at 25–30 g/L concentration from enzymatically hydrolysed soy-hull using *Saccharomyces cerevisiae*. Three enzymes, namely pectinase, β-glucosidase, and cellulase were used to hydrolysed soy-hull before fermentation. Another research exploited soy-hull as a fermentation medium for enzyme production. Cellulases and xylanases were effectively produced from soy-hull using *Trichoderma reesei* (122). Besides, high production of peptidases (1,000 ± 100 AU/mL) was obtained when soy-hull were mixed with orange peel as substrates for *Aspergillus niger* NRRL 3 fermentation. As the interest in enzyme production from microbial sources has shown great development, soy-hull posts a potential low-cost substrate for enzyme production.

It is important to highlight that some methods are used to overcome the drawbacks of the soy protein-based adhesive, which are low water resistance and adhesive strength. The methods used to modify the soy adhesive, include Primary Secondary 3 cross-linking, chemical denaturation, hydrolysis, enzyme modification, and others (123). Besides, antinutritional factors such as trypsin inhibitors in soybean could be one of the limiting factors of soybean waste as it will prevent protein digestion. However, simple treatments such as soaking and heating manage to inactivate the inhibitors (124).

## 5. Limitations of study and areas of further studies

### 5.1. Limitations of the study

The limitations of this study are inherent to the method since narrative reviews do not follow a systematic approach, not being replicable. However, narrative literature review articles are essential in continuing education because they provide readers with up-to-date knowledge about a specific topic, in which relevant studies’ findings are discussed to present an argument about the conclusions drawn from the current state of knowledge. However, as the main characteristics, this type of review does not describe the methodological approach nor answer specific quantitative research questions. Therefore, the potential limitation of the method is, although the reviewers will learn about the problem, they will not arrive at a comprehensive understanding of the state of the science related to the problem.

### 5.2. Improvements and areas of further studies

The current agriculture and food systems are at risk due to the urgent issues of the present and the future, including the struggle for natural resources, population growth, food losses and waste, and climate change. These elements necessitate a change in our agricultural landscape and exposure to more sustainable food production processes and products. For long-term prospects and future food supplies, more focus must be placed on the creation of various and alternative functional foods and other possible products employing mushroom, peanut, and soybean wastes and increasing dietary diversity, including these food groups.

## 6. Conclusion

Recycling wastes and byproducts from food processing industries has become a desirable alternative to disposal because of environmental awareness and to ensure food sustainability. Thus, this review highlights the potential reutilisation of high protein materials from mushroom, peanut, and soybean (MPS) as functional components in health, nutraceuticals, and food applications.

Mushrooms are important dietary food item, tonics, medicines, and sources of high nutrition. Mushrooms are low in fat, calories, and carbs and high in protein, crude fiber, minerals, and vitamins. They offer superior carbohydrates that improve human health. Mushrooms are used as a meat substitute because of their nutritional content, which is on par with many vegetables. Growing mushrooms is an activity that can turn trash into the best-nutritional food with a high protein conversion efficiency.

The harvesting and crushing of peanuts result in the production of byproducts such as peanut meal, peanut skin, peanut hull, and peanut vine. Protein, fiber, and polyphenols are just a few of the valuable substances found in peanut byproducts that can be used as functional additives in processed meals. The food processing sector may use some waste and byproducts from the peanut enterprise.

Soybean provides a superior source of plant protein with a well-proportioned essential amino acid. Its cultivation is significant from an economic standpoint. During the manufacturing of primary soy-based goods, a large amount of byproducts such as okara, soybean meal, soy-hull and soy-whey are produced together. These organic wastes contain a high amount of organic and bioactive compounds that could further be reutilized in food and non-food applications. However, further research on the appropriate method of waste reutilization as food ingredients or non-food-based material synthesis is needed.

## Author contributions

NM, MA, and WW-M: conceptualization, project administration, writing—original draft preparation, and visualization. WW-M: methodology and investigation. NA: software. AJ and AR: validation. NA and WW-M: formal analysis. AR: resources. SR and NA: data curation. SR, NM, MA, AR, RZ, MB, DR, LL, HH, and WW-M: writing—review and editing. WW-M and AJ: supervision. AR, LL, HH, and MB: funding acquisition. All authors have read and agreed to the published version of the manuscript.

## Funding

This research was funded by Fundamental Research Grant (FRGS) Grant no. 01-01-20-2323FR, with reference code: FRGS/1/2020/STG01/UPM/02/2 and UM International collaboration grant, ST002-2022.

## Conflict of interest

The authors declare that the research was conducted in the absence of any commercial or financial relationships that could be construed as a potential conflict of interest.

## Publisher’s note

All claims expressed in this article are solely those of the authors and do not necessarily represent those of their affiliated organizations, or those of the publisher, the editors and the reviewers. Any product that may be evaluated in this article, or claim that may be made by its manufacturer, is not guaranteed or endorsed by the publisher.
