# Supplementary material for: A narrative action on the battle against hunger using mushroom, peanut, and soybean-based wastes
Source: Front Public Health. 2023 May 11;11:1175509. doi: 10.3389/fpubh.2023.1175509 (PMC10213758; doi:10.3389/fpubh.2023.1175509)
Supplement: Supplementary file 1 [file Data_Sheet_1.docx]

Supplementary Material

A Narrative Action on the Battle against Hunger using Mushroom, Peanut, and Soybean-based Wastes

**Nurul Aqilah Mohd Zaini ^1^, Nur Asyiqin Zahia Azizan^2^, Muhamad Hafiz Abd Rahim^3*^, Adi Ainurzaman Jamaludin^4^, António Raposo ^5^, Siva Raseetha^6^, Renata Puppin Zandonadi ^7^, Mona N. BinMowyna^8^, Dele Raheem ^9^, Linda Heejung Lho ^10*^, Heesup Han ^11*^, and** **Wan Abd Al Qadr Imad Wan-Mohtar^2*^**

^1^Department of Food Sciences, Faculty of Science and Technology, Universiti Kebangsaan Malaysia, 43600 Bangi, Selangor, Malaysia; [nurulaqilah@ukm.edu.my](mailto:nurulaqilah@ukm.edu.my)

^2^ Functional Omics and Bioprocess Development Laboratory, Institute of Biological Sciences, Faculty of Science, Universiti Malaya, 50603 Kuala Lumpur, Malaysia

^3^Faculty of Food Science and Technology, Universiti Putra Malaysia, 43400 Serdang, Selangor, Malaysia

^4^Environmental Management Program, Institute of Biological Sciences, Faculty of Science, Universiti Malaya, 50603 Kuala Lumpur, Malaysia; [adiainurzaman@um.edu.my](mailto:adiainurzaman@um.edu.my)

^5^CBIOS (Research Center for Biosciences and Health Technologies), Universidade Lusófona de Humanidades e Tecnologias, Campo Grande 376, 1749-024 Lisboa, Portugal

^6^ Faculty of Applied Sciences, Universiti Teknologi MARA, 40450 Shah Alam, Selangor, Malaysia

^7^Department of Nutrition, University of Brasília, Brasília 70910-900, Brazil

^8^College of Applied Medical Sciences, Shaqra University, Shaqra 11961, Saudi Arabia

^9^Northern Institute for Environmental and Minority Law (NIEM), Arctic Centre, University of Lapland, 96101 Rovaniemi, Finland

^10^ College of Business Division of Tourism and Hotel Management, Cheongju University, 298 Daesung-ro, Cheongwon-gu, Cheongju-si, Chungcheongbuk-do, 28503, Republic of Korea

^11^ College of Hospitality and Tourism Management, Sejong University, 98 Gunja-Dong, Gwanjin-Gu, Seoul 143-747 Republic of Korea

**Correspondence:**

[muhdhafiz@upm.edu.my](mailto:muhdhafiz@upm.edu.my), [heeelho@gmail.com](mailto:heeelho@gmail.com), [heesup.han@gmail.com](mailto:heesup.han@gmail.com), [qadyr@um.edu.my](mailto:qadyr@um.edu.my)

# Supplementary Figures and Tables

## Supplementary Figures

##
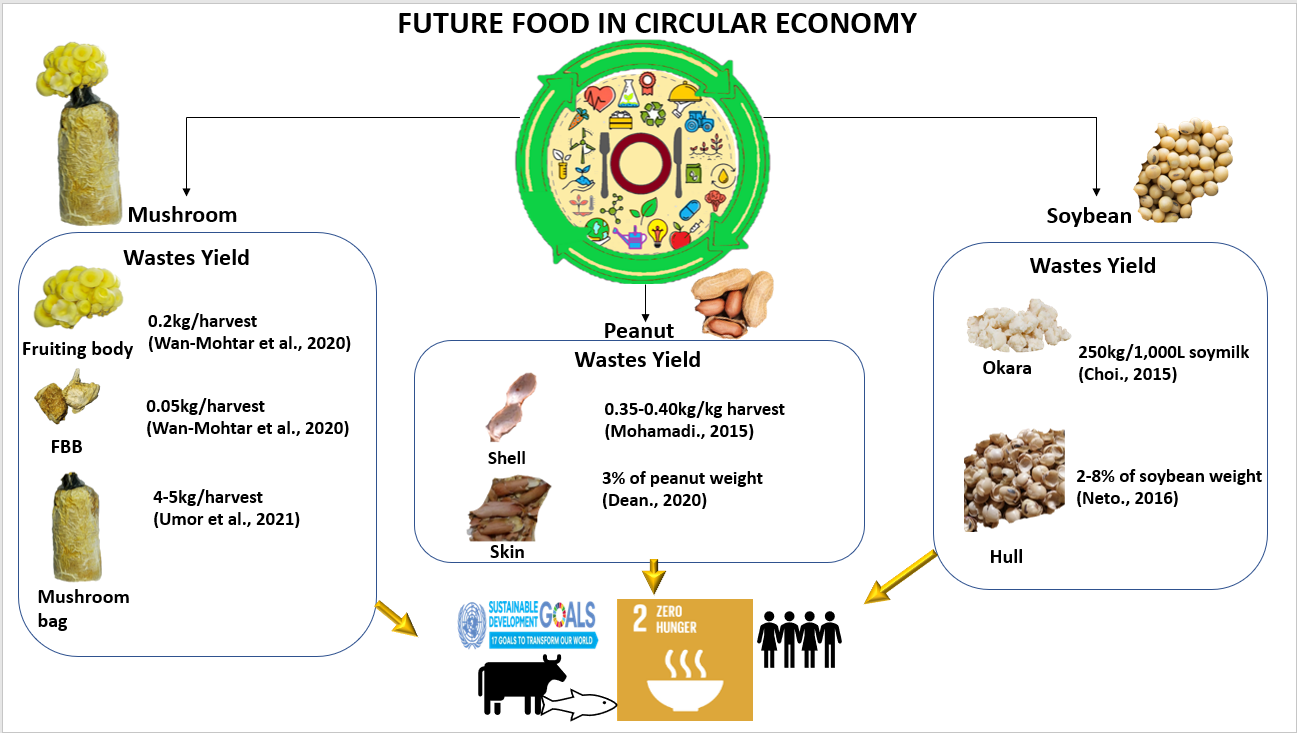


## Supplementary Figure 1. Future food in circular economy of mushroom, peanut, and soybean (MPS) wastes towards achieving SDG2 - Zero hunger


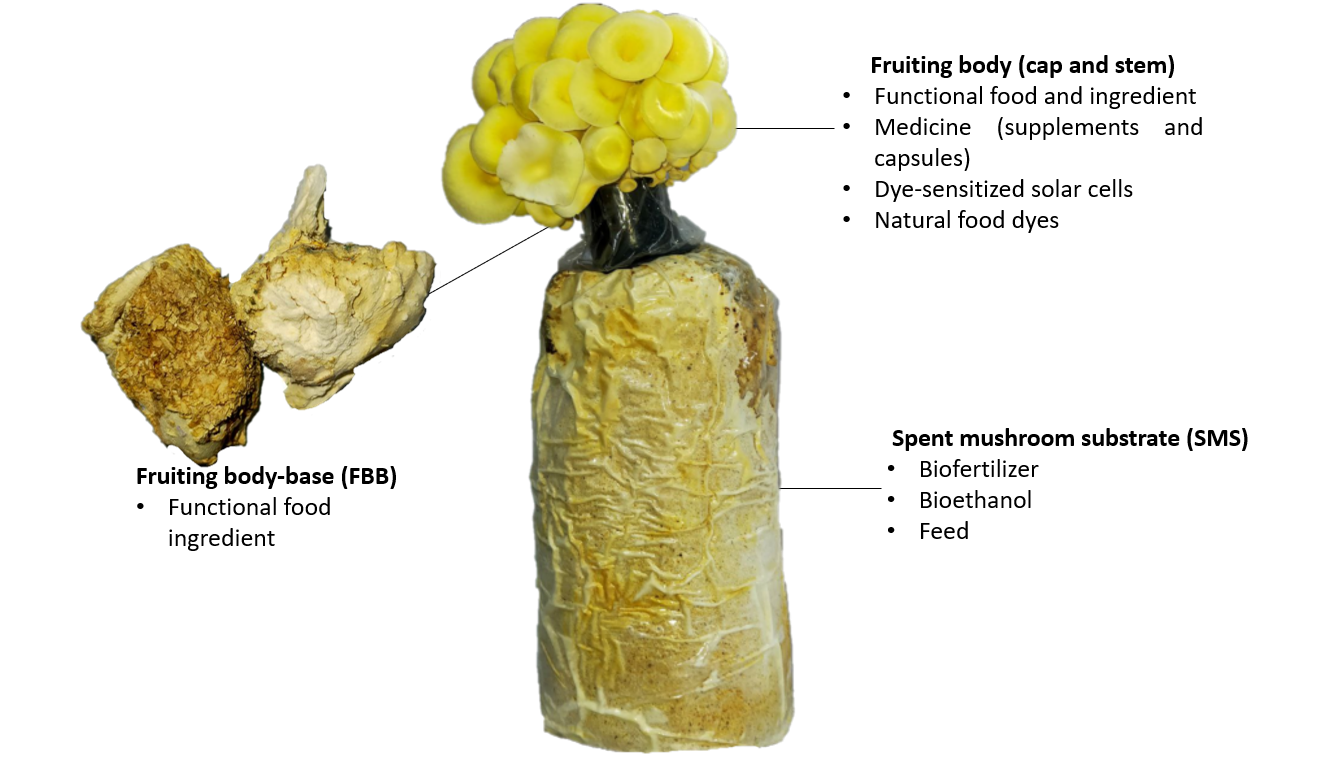


**Supplementary Figure 2.** Parts of usable mushroom biomass and spent SMS.


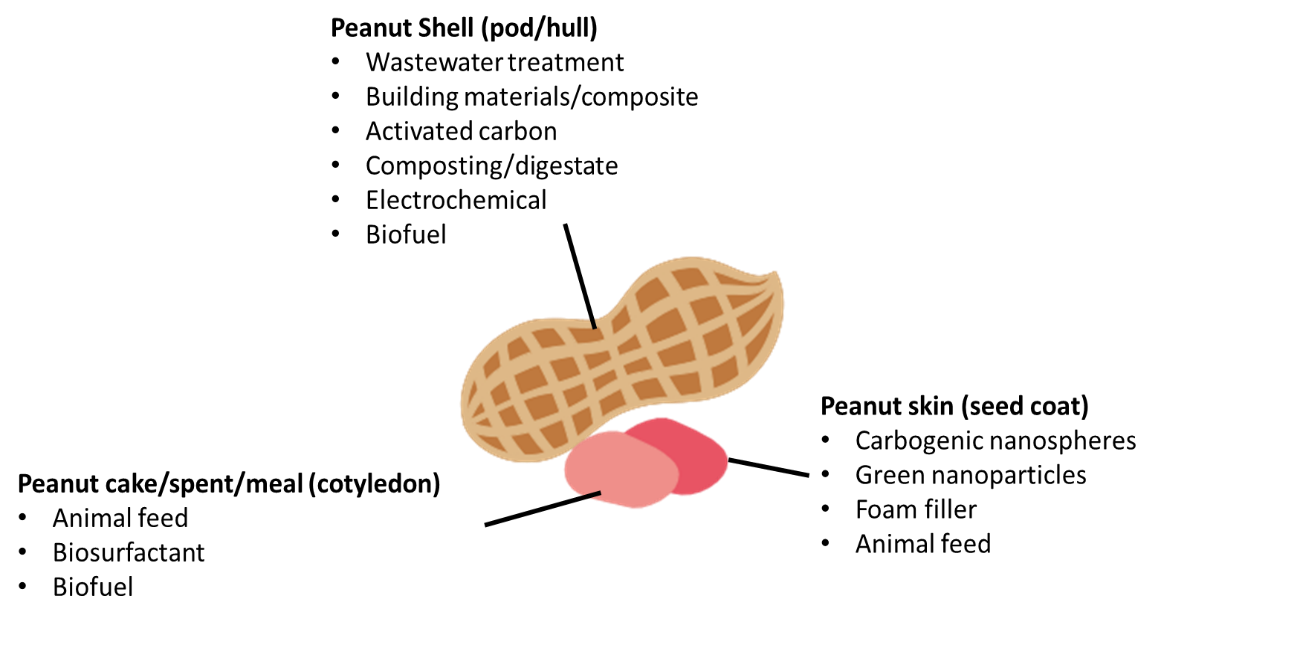


**Supplementary Figure 3**. The three major waste of peanut processing and their non-food applications. The kernels are the main component used in the food industry, while the shell, spent (meal or cake),

and skin are typically discarded as by-products. (Adapted from (Duc et al., 2019), (Toomer, 2020), and (Sorita et al., 2020).

**
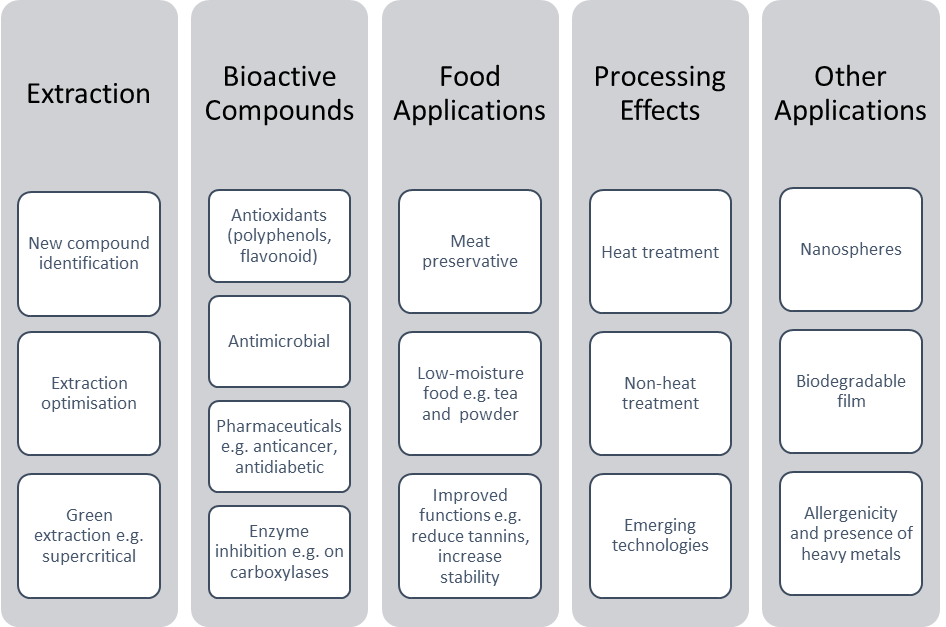
**

**Supplementary Figure 4.** The current area of food research on peanut skin. (Adapted from (Dean et al., 2008, Lorenzo et al., 2018, Sorita et al., 2020).


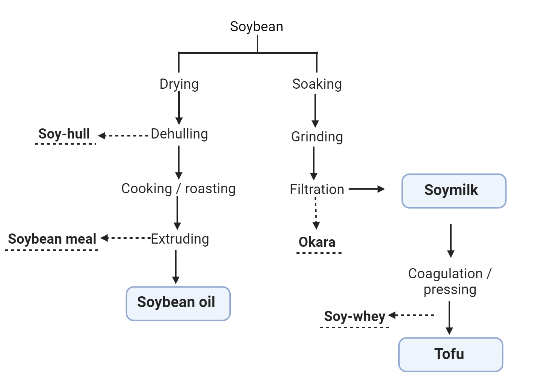


**Supplementary Figure 5.** An illustration of the soymilk production procedures and the waste products produced by the process.

## Supplementary Tables

Supplementary Table 1. Recent applications and products by part or waste of respective mushroom species

| Mushroom species | Part used | Application and product | References |
| --- | --- | --- | --- |
| Oyster mushroom (*Pleurotus sapidus*) | FBB | Antioxidative flour in the cookie and steamed bun | (Wan-Mohtar et al., 2018) |
| Grey oyster (*Pleurotus sajur-caju*) mushroom stems | Stem | Meat replacer in chicken nuggets | (Husain and Huda-Faujan, 2020) |
| Oyster mushroom (*Pleurotus sapidus*) | FBB | Antioxidative flour in chicken patty | (Wan‐Mohtar et al., 2020) |
| *Ganoderma lucidum* | SMS | Bioethanol | (Sudhakar et al., 2021) |
| Enoki mushroom  (*Flammulina*  *Velutipes*) | Mushroom stem waste (MSW) | Functional powder in goat meat nuggets | (Banerjee et al., 2020) |
| *Lentinus edodes*,  *Pleurotus ostreatus* and *coprinus comatus* | Fruiting body | Food ingredient and additive replacer | (Yuan et al., 2021) |
| *Pleurotus ostreatus* | SMS | Biogas | (Gao et al., 2021) |
| Oyster mushroom | Fruiting body | Natural food dye and sensitized solar cells | (Zhang et al., 2022, Ahmad et al., 2022) |

**Supplementary Table 2**. The potential use of peanut shell or its extract in food-related applications.

| Application | Process | Remarks | References |
| --- | --- | --- | --- |
| Production of monophenolic compounds | Lignin depolymerisation through fractionation, depolymerization, and separation/purification. | Fractionation includes hot compressed water, solvent, and catalytic fractionation. Depolymerisation with solvent is assisted by microwave | (Routray et al., 2022) |
| Synthesis of silver nanoparticles | Production of extract followed by addition of silver nitrate | Comparable to the control and possess antifungal activity | (Velmurugan et al., 2015) |
| Extraction of polyphenols | Microwave and enzymatic treatment with cellulase | The microwave-assisted enzymatic extraction method is better than heat, sonication and enzymatic treatments and the polyphenols have good antioxidant and antibacterial properties | (Zhang et al., 2013) |
| Starch-chitosan film (active packaging) | Drying and filtration | The antioxidative capability of peanut skin is higher than that of the shell, but both decreased the swelling and water vapour permeability of the film. | (Meng et al., 2020, Zhang et al., 2020) |
| Extraction of bioactive oligosaccharides | Autohydrolysis (non-isothermal aqueous) and alkaline extraction | Breakdown of hemicellulose produced soluble oligosaccharides (xylose polymer with acetyl and methylglucuronosyl groups) and xyloglucan with high antioxidant ability | (Arumugam et al., 2018, Rico et al., 2018) |
| Extraction of flavonoids | Ultrasonic-assisted process (UAP) | UAE produced higher flavonoids than Soxhlet or heat-assisted extraction | (Liao et al., 2021) |
| Safety assessment *in vivo* | Rats toxicological test | Polyphenols from the shells are non-toxic as it causes no mutations and changes in body weight and clinical signs | (Gao et al., 2011) |
| Antidiabetic properties *in vitro* and *in vivo* | UAE with response surface methodology and *in vivo* | α-glucosidase and pancreatic lipase inhibitory effects *in vitro* and improve diabetic marker in rats | (Gam et al., 2021, Sun et al., 2018) |
| Neurotrophic function *in vitro* | *In vitro* assessment of neuron-related signalling and cells | The phytochemicals in the shells stimulate transcription factors in neuron cells | (Gao et al., 2022) |
| Protein-rich substrate | Fermentation with sweet potato beverage residues by *Aspergillus oryzae* and *Bacillus subtilis* | Increasing the protein components in sweet potato waste | (Zuo et al., 2018) |
| Hypoxanthine-xanthine oxidase-induced injury *in vitro* | Erythrocyte model | Luteolin protects erythrocytes morphology and function and reduces the uric acid formation | (Peng et al., 2021) |
| Degradation of peanut shell biomass | Submerged fermentation by *Inonotus obliquus* | Lignocellulose degradation produced high biomass, production of antioxidants, polysaccharides, and polyphenols | (Xu et al., 2014) |

**Supplementary Table 3**. The recent use of peanut cake in food or pharmaceutical applications

| Food Product | Benefits | References |
| --- | --- | --- |
| Fermented press cake | *Aspergillus* spp. increased phenolic content, antioxidants, functional properties, microstructural protein content, and sensorial qualities | (Sadh et al., 2018) |
|  | Lipopeptide from Bacillus cereus SNAU01 was produced and characterised under solid-state fermentation, and it may be used as an anti-biofilm agent. | (Nalini et al., 2016) |
|  | *Bacillus* spp. fermentation increases the degree of hydrolysis, antioxidant capacities, and improved flavor profiles of peanut meal peptides | (Qinzhu et al., 2018, Yang et al., 2016) |
|  | Bacterial (*Sporolactobacillus*) fermentation and enzymatic hydrolysis produced higher D-lactate compared to other nitrogen sources | (Xiang et al., 2016) |
|  | Bacterial (*Streptococcus*) and yeast (*Zygosaccharomyces*) fermentation degraded cancerous aflatoxin B_1_ and G_1_ | (Chen et al., 2015, Zhao et al., 2017) |
|  | Bacterial fermentation improves gut microbiota balance and brain function *in vivo* | (Jiang et al., 2020) |
| Defatted peanut cake | Optimal extraction parameters yielded 25.89% with primary polysaccharides being α-galactose | (Song et al., 2011) |
|  | Alcalase and pepsin produce hydrolysates with varying degrees of solubility, emulsification abilities, fat absorption capacity, and antioxidative ability | (Nyo and Nguyen, 2019) |
|  | Isolation, solubilisation, and emulsification of arachin protein using ultra-high-pressure processing | (Zhao et al., 2015) |
|  | A polysaccharide (2.383 105 Da) made of glucose, galactose, arabinose, and xylose was extracted, purified, and characterised using hot water. | (Liu et al., 2016) |
|  | Ultrasonic-assisted enzymolysis improve the DPPH antioxidant activities up to 90.06% (and 7 other antioxidant parameters) compared to single protease hydrolysis | (Yu et al., 2012) |
|  | Peanut meal flour that has been partially de-oiled improves the bread's nutrient, sensory, and textural qualities. | (Yadav et al., 2013) |
|  | Optimisation of an extrusion process to create alternative meat by exploiting the arachin fibrous properties | (Zhang et al., 2019) |

**Supplementary Table 4.** Potential functional properties and health benefits of soybean waste for food applications

| Soybean waste | Food application | Functional properties / health benefits | References |
| --- | --- | --- | --- |
| Okara | Yogurt | Firmness - Increase the firmness of yogurt | (Bedani et al., 2014) |
|  | - | Prebiotic - enhanced short-chain fatty acids (SCFA) release | (Pérez-López et al., 2018) |
|  | - | Enhanced absorption of calcium and magnesium |  |
|  | Ice cream | Prebiotics - support the growth of *Lactobacillus plantarum* | (Ibrahim et al., 2022) |
|  |  | Slow melting rate |  |
|  | Gluten-free cookies | Increased protein and fiber content, increased hardness | (Ostermann-Porcel et al., 2017) |
|  | Butter cake | More ability to absorb water and oil, as well as more protein, fibre, and total phenolic content | (Nguyen Doan Mai et al., 2021) |
|  | Sausage | Increased water holding capacity, protein solubility, emulsion stability, and emulsification capability | (Grizotto et al., 2012) |
| Enzymatically- hydrolysed soybean meal | Biscuits | Dietary fibre sources, protein sources, phosphorus, potassium, calcium, zinc, copper, iron, manganese, and magnesium sources | (Barreto et al., 2022) |
| Fermented soybean meal and soybean meal and | Biscuits | Low carbohydrate, high fiber, high protein, high isoflavones (antioxidant) | (Silva et al., 2018) |
| Soy-whey | Functional probiotic soy drink | Enrichment in bioactive isoflavone aglycones  Support the growth of *Lactobacillus rhamnosus* GG and *Lactobacillus paracasei* | (Zhu et al., 2019) |
|  | Soy alcoholic beverages | Changes the flavour profile soy-whey  Increase the antioxidant properties by hydrolysing isoflavone glucosides to isoflavone aglycones. | (Chua et al., 2018) |

**References**

Ahmad, N., Vunduk, J., Klaus, A., Dahlan, N. Y., Ghosh, S., Muhammad-Sukki, F., Dufossé, L., Bani, N. A. & Wan-Mohtar, W. A. A. Q. I. 2022. Roles of medicinal mushrooms as natural food dyes and dye-sensitised solar cells (DSSC): synergy of zero hunger and affordable energy for sustainable development. *Sustainability,* 14**,** 13894. <https://doi.org/10.3390/su142113894>

Arumugam, N., Biely, P., Puchart, V., Singh, S. & Pillai, S. 2018. Structure of peanut shell xylan and its conversion to oligosaccharides. *Process Biochemistry,* 72**,** 124-129. <https://doi.org/10.1016/j.procbio.2018.06.024>

Banerjee, D. K., Das, A. K., Banerjee, R., Pateiro, M., Nanda, P. K., Gadekar, Y. P., Biswas, S., Mcclements, D. J. & Lorenzo, J. M. 2020. Application of enoki mushroom (*Flammulina Velutipes*) stem wastes as functional ingredients in goat meat nuggets. *Foods,* 9**,** 432. <https://doi.org/10.3390/foods9040432>

Barreto, N. M. B., Sandôra, D., Braz, B. F., Santelli, R. E., De Oliveira Silva, F., Monteiro, M. & Perrone, D. 2022. Biscuits Prepared with Enzymatically-Processed Soybean Meal Are Rich in Isoflavone Aglycones, Sensorially Well-Accepted and Stable during Storage for Six Months. *Molecules,* 27**,** 7975.  <https://doi.org/10.3390/molecules27227975>

Bedani, R., Campos, M. M., Castro, I. A., Rossi, E. A. & Saad, S. M. 2014. Incorporation of soybean by‐product okara and inulin in a probiotic soy yoghurt: texture profile and sensory acceptance. *Journal of the Science of Food and Agriculture,* 94**,** 119-125. <https://doi.org/10.1002/jsfa.6212>

Chen, Y., Kong, Q., Chi, C., Shan, S. & Guan, B. 2015. Biotransformation of aflatoxin B1 and aflatoxin G1 in peanut meal by anaerobic solid fermentation of *Streptococcus thermophilus* and *Lactobacillus delbrueckii subsp. bulgaricus*. *International journal of food microbiology,* 211**,** 1-5. <https://doi.org/10.1016/j.ijfoodmicro.2015.06.021>

Choi, I. S., Kim, Y. G., Jung, J. K. & Bae, H.-J. 2015. Soybean Waste (Okara) As A Valorization Biomass For The Bioethanol Production. Energy, 93, 1742-1747. <https://doi.org/10.1016/j.energy.2015.09.093>

Chua, J.-Y., Lu, Y. & Liu, S.-Q. 2018. Evaluation of five commercial non-Saccharomyces yeasts in fermentation of soy (tofu) whey into an alcoholic beverage. *Food Microbiology,* 76**,** 533-542. <https://doi.org/10.1016/j.fm.2018.07.016>

Dean, L., Davis, J., Shofran, B. & Sanders, T. 2008. Phenolic profiles and antioxidant activity of extracts from peanut plant parts. *The Open Natural Products Journal,* 1. [**DOI:**[10.2174/1874848100801010001](http://dx.doi.org/10.2174/1874848100801010001)]

Dean, L.L., 2020. Extracts of peanut skins as a source of bioactive compounds: Methodology and applications. *Applied Sciences*, *10*(23), p.8546. <https://doi.org/10.3390/app10238546>

Duc, P., Dharanipriya, P., Velmurugan, B. & Shanmugavadivu, M. 2019. Groundnut shell-a beneficial bio-waste. *Biocatal Agric Biotechnol* 20: 101206. <https://doi.org/10.1016/j.bcab.2019.101206>

Gam, D. H., Hong, J. W., Yeom, S. H. & Kim, J. W. 2021. Polyphenols in peanut shells and their antioxidant activity: optimal extraction conditions and the evaluation of anti-obesity effects. *Journal of Nutrition and Health,* 54**,** 116-128. <https://doi.org/10.4163/jnh.2021.54.1.116>

Gao, A. X., Xiao, J., Xia, T. C.-X., Dong, T. T.-X. & Tsim, K. W.-K. 2022. The extract of peanut shell enhances neurite outgrowth of neuronal cells: Recycling of agricultural waste for development of nutraceutical products. *Journal of Functional Foods,* 91**,** 105023. <https://doi.org/10.1016/j.jff.2022.105023>

Gao, F., Ye, H., Yu, Y., Zhang, T. & Deng, X. 2011. Lack of toxicological effect through mutagenicity test of polyphenol extracts from peanut shells. *Food chemistry,* 129**,** 920-924. <https://doi.org/10.1016/j.foodchem.2011.05.046>

Gao, X., Tang, X., Zhao, K., Balan, V. & Zhu, Q. 2021. Biogas production from anaerobic co-digestion of spent mushroom substrate with different livestock manure. *Energies,* 14**,** 570. <https://doi.org/10.3390/en14030570>

Grizotto, R. K., Andrade, J. C. D., Miyagusku, L. & Yamada, E. A. 2012. Physical, chemical, technological and sensory characteristics of Frankfurter type sausage containing okara flour. *Food Science and Technology,* 32**,** 538-546. <https://doi.org/10.1590/S0101-20612012005000076>

Husain, H. & Huda-Faujan, N. 2020. Potential application of grey oyster mushroom stems as halal meat replacer in imitation chicken nuggets. *Food Research*. [10.26656/fr.2017.4(S1).S18](https://doi.org/10.26656/fr.2017.4(S1).S18)

Ibrahim, I. N., Kamaruding, N. A., Ismail, N. & Shaharuddin, S. 2022. Value addition to ice cream by fortification with okara and probiotic. *Journal of Food Processing and Preservation,* 46**,** e16253. <https://doi.org/10.1111/jfpp.16253>

Jiang, X., Ding, H., Liu, Q., Wei, Y., Zhang, Y., Wang, Y., Lu, Y., Ma, A., Li, Z. & Hu, Y. 2020. Effects of peanut meal extracts fermented by Bacillus natto on the growth performance, learning and memory skills and gut microbiota modulation in mice. *British Journal of Nutrition,* 123**,** 383-393. <https://doi.org/10.1017/S0007114519002988>

Liao, J., Guo, Z. & Yu, G. 2021. Process intensification and kinetic studies of ultrasound-assisted extraction of flavonoids from peanut shells. *Ultrasonics Sonochemistry,* 76**,** 105661. <https://doi.org/10.1016/j.ultsonch.2021.105661>

Liu, H., Jiang, N., Liu, L., Sheng, X., Shi, A., Hu, H., Yang, Y. & Wang, Q. 2016. Extraction, purification and primary characterization of polysaccharides from Defatted Peanut (Arachis hypogaea) Cakes. *Molecules,* 21**,** 716. <https://doi.org/10.3390/molecules21060716>

Lorenzo, J. M., Munekata, P. E., Sant'ana, A. S., Carvalho, R. B., Barba, F. J., Toldrá, F., Mora, L. & Trindade, M. A. 2018. Main characteristics of peanut skin and its role for the preservation of meat products. *Trends in Food Science & Technology,* 77**,** 1-10. <https://doi.org/10.1016/j.tifs.2018.04.007>

Mohammadi Torkashvand, A., Alidoust, M. and Mahboub Khomami, A., 2015. The reuse of peanut organic wastes as a growth medium for ornamental plants. *International Journal of Recycling of Organic Waste in Agriculture*, *4*, pp.85-94. https://doi.org/10.1007/s40093-015-0088-0<https://doi.org/10.1007/s40093-015-0088-0>

Meng, W., Shi, J., Zhang, X., Lian, H., Wang, Q. & Peng, Y. 2020. Effects of peanut shell and skin extracts on the antioxidant ability, physical and structure properties of starch-chitosan active packaging films. *International journal of biological macromolecules,* 152**,** 137-146. <https://doi.org/10.1016/j.ijbiomac.2020.02.235>

Nalini, S., Parthasarathi, R. & Prabudoss, V. 2016. Production and characterization of lipopeptide from *Bacillus cereus* SNAU01 under solid state fermentation and its potential application as anti-biofilm agent. *Biocatalysis and Agricultural Biotechnology,* 5**,** 123-132. <https://doi.org/10.1016/j.bcab.2016.01.007>

Neto, W.P.F., Mariano, M., da Silva, I.S.V., Silvério, H.A., Putaux, J.L., Otaguro, H., Pasquini, D. and Dufresne, A., 2016. Mechanical properties of natural rubber nanocomposites reinforced with high aspect ratio cellulose nanocrystals isolated from soy hulls. *Carbohydrate polymers*, *153*, pp.143-152. <https://doi.org/10.1016/j.carbpol.2016.07.073>

Nguyen Doan Mai, H., Phan Thi Lan, K., Techapun, C., Leksawasdi, N., Taesuwan, S., Hanprom, N., Sompakdee, N., Nunta, R. & Khemacheewakul, J. 2021. Quality Evaluation of Butter Cake Prepared by Substitution of Wheat Flour with Green Soybean (*Glycine Max L.*) Okara. *Journal of Culinary Science & Technology***,** 1-14. <https://doi.org/10.1080/15428052.2021.1978363>

Nyo, M. K. & Nguyen, L. T. 2019. Value-addition of defatted peanut cake by proteolysis: effects of proteases and degree of hydrolysis on functional properties and antioxidant capacity of peptides. *Waste and Biomass Valorization,* 10**,** 1251-1259. <https://doi.org/10.1007/s12649-017-0146-0>

Ostermann-Porcel, M. V., Quiroga-Panelo, N., Rinaldoni, A. N. & Campderrós, M. E. 2017. Incorporation of Okara into Gluten-Free Cookies with High Quality and Nutritional Value. *Journal of Food Quality,* 2017**,** 4071585. <https://doi.org/10.1155/2017/4071585>

Peng, M., Chen, Z., Deng, Q., Zhu, S. & Wang, G. 2021. The roles of luteolin in peanut shell extract-Mediated protection of erythrocytes against hypoxanthine-xanthine oxidase-induced toxicity. *Food Bioscience,* 39**,** 100826. <https://doi.org/10.1016/j.fbio.2020.100826>

Pérez-López, E., Veses, A. M., Redondo, N., Tenorio-Sanz, M. D., Villanueva, M. J., Redondo-Cuenca, A., Marcos, A., Nova, E., Mateos-Aparicio, I. & Rupérez, P. 2018. Soybean Okara modulates gut microbiota in rats fed a high-fat diet. *Bioactive Carbohydrates and Dietary Fibre,* 16**,** 100-107. <https://doi.org/10.1016/j.bcdf.2018.09.002>

Qinzhu, Z., Yan-Ling, C., Dong-Xiao, S., Tian, B., Yang, Y. & Shan, H. 2018. Process optimization and anti-oxidative activity of peanut meal Maillard reaction products. *Lwt,* 97**,** 573-580. <https://doi.org/10.1016/j.lwt.2018.07.025>

Rico, X., Gullon, B., Alonso, J. L., Parajó, J. C. & Yanez, R. 2018. Valorization of peanut shells: Manufacture of bioactive oligosaccharides. *Carbohydrate polymers,* 183**,** 21-28. <https://doi.org/10.1016/j.carbpol.2017.11.009>

Routray, W., Jena, B. & Orsat, V. 2022. Recent advances in extraction, isolation, characterization, and applications of phenolic compounds. *Studies in Natural Products Chemistry,* 72**,** 29-55. <https://doi.org/10.1016/B978-0-12-823944-5.00006-5>

Sadh, P. K., Chawla, P. & Duhan, J. S. 2018. Fermentation approach on phenolic, antioxidants and functional properties of peanut press cake. *Food bioscience,* 22**,** 113-120. <https://doi.org/10.1016/j.fbio.2018.01.011>

Silva, F. D. O., Miranda, T. G., Justo, T., Frasão, B. D. S., Conte-Junior, C. A., Monteiro, M. & Perrone, D. 2018. Soybean meal and fermented soybean meal as functional ingredients for the production of low-carb, high-protein, high-fiber and high isoflavones biscuits. *LWT,* 90**,** 224-231. <https://doi.org/10.1016/j.lwt.2017.12.035>

Song, Y., Du, B., Zhou, T., Han, B., Yu, F., Yang, R., Hu, X., Ni, Y. & Li, Q. 2011. Optimization of extraction process by response surface methodology and preliminary structural analysis of polysaccharides from defatted peanut (*Arachis hypogaea*) cakes. *Carbohydrate research,* 346**,** 305-310. <https://doi.org/10.1016/j.carres.2010.11.019>

Sorita, G. D., Leimann, F. V. & Ferreira, S. R. S. 2020. Biorefinery approach: is it an upgrade opportunity for peanut by-products? *Trends in Food Science & Technology,* 105**,** 56-69. <https://doi.org/10.1016/j.tifs.2020.08.011>

Sudhakar, M., Ravel, M. & Perumal, K. 2021. Pretreatment and process optimization of bioethanol production from spent biomass of Ganoderma lucidum using *Saccharomyces cerevisiae*. *Fuel,* 306**,** 121680. <https://doi.org/10.1016/j.fuel.2021.121680>

Sun, X.-M., Ye, H.-Q., Liu, J.-B., Wu, L., Lin, D.-B., Yu, Y.-L. & Gao, F. 2018. Assessment of anti-diabetic activity of peanut shell polyphenol extracts. *Journal of Zhejiang University-SCIENCE B,* 19**,** 764-775. <https://doi.org/10.1631/jzus.B1700401>

Toomer, O. T. 2020. A comprehensive review of the value-added uses of peanut (*Arachis hypogaea*) skins and by-products. *Critical reviews in food science and nutrition,* 60**,** 341-350. <https://doi.org/10.1080/10408398.2018.1538101>

Umor, N.A., Ismail, S., Abdullah, S., Huzaifah, M.H.R., Huzir, N.M., Mahmood, N.A.N. and Zahrim, A.Y., 2021. Zero waste management of spent mushroom compost. *Journal of Material Cycles and Waste Management*, *23*(5), pp.1726-1736. <https://doi.org/10.1007/s10163-021-01250-3>

Velmurugan, P., Sivakumar, S., Young-Chae, S., Seong-Ho, J., Pyoung-In, Y., Jeong-Min, S. & Sung-Chul, H. 2015. Synthesis and characterization comparison of peanut shell extract silver nanoparticles with commercial silver nanoparticles and their antifungal activity. *Journal of Industrial and Engineering Chemistry,* 31**,** 51-54. <https://doi.org/10.1016/j.jiec.2015.06.031>

Wan-Mohtar, W., Mahmud, N., Supramani, S., Ahmad, R., Zain, N. A. M., Hassan, N. A., Peryasamy, J. & Halim-Lim, S. A. 2018. Fruiting-body-base flour from an oyster mushroom-a waste source of antioxidative flour for developing potential functional cookies and steamed-bun. *AIMS Agriculture and Food*. doi: [10.3934/agrfood.2018.4.481](https://doi.org/10.3934/agrfood.2018.4.481)

Wan‐Mohtar, W. A. A. Q. I., Halim‐Lim, S. A., Kamarudin, N. Z., Rukayadi, Y., Abd Rahim, M. H., Jamaludin, A. A. & Ilham, Z. 2020. Fruiting‐body‐base flour from an Oyster mushroom waste in the development of antioxidative chicken patty. *Journal of food science,* 85**,** 3124-3133. <https://doi.org/10.1111/1750-3841.15402>

Xiang, L., Cao, X.-L., Xing, T.-Y., Mori, D., Tang, R.-Q., Li, J., Gao, L.-J. & Qi, J.-H. 2016. Mixture of peanut skin extract and fish oil improves memory in mice via modulation of anti-oxidative stress and regulation of BDNF/ERK/CREB signaling pathways. *Nutrients,* 8**,** 256. <https://doi.org/10.3390/nu8050256>

Xu, X.-Q., Hu, Y. & Zhu, L.-H. 2014. The capability of Inonotus obliquus for lignocellulosic biomass degradation in peanut shell and for simultaneous production of bioactive polysaccharides and polyphenols in submerged fermentation. *Journal of the Taiwan Institute of Chemical Engineers,* 45**,** 2851-2858. <https://doi.org/10.1016/j.jtice.2014.08.029>

Yadav, D., Thakur, N., Sunooj, K. & Singh, K. 2013. Effect of de-oiled peanut meal flour (DPMF) on the textural, organoleptic and physico chemical properties of bread. *International Food Research Journal,* 20**,** 1307. [DOI:10.4236/fns.2012.34067](http://www.scirp.org/journal/PaperInformation.aspx?PaperID=18490)

Yang, X., Teng, D., Wang, X., Guan, Q., Mao, R., Hao, Y. & Wang, J. 2016. Enhancement of nutritional and antioxidant properties of peanut meal by bio-modification with *Bacillus licheniformis*. *Applied biochemistry and biotechnology,* 180**,** 1227-1242. <https://doi.org/10.1007/s12010-016-2163-z>

Yu, L., Sun, J., Liu, S., Bi, J., Zhang, C. & Yang, Q. 2012. Ultrasonic-assisted enzymolysis to improve the antioxidant activities of peanut (*Arachin conarachin L*.) antioxidant hydrolysate. *International journal of molecular sciences,* 13**,** 9051-9068. <https://doi.org/10.3390/ijms13079051>

Yuan, X., Jiang, W., Zhang, D., Liu, H. & Sun, B. 2021. Textural, sensory and volatile compounds analyses in formulations of sausages analogue elaborated with edible mushrooms and soy protein isolate as meat substitute. *Foods,* 11**,** 52. <https://doi.org/10.3390/foods11010052>

Zhang, G., Hu, M., He, L., Fu, P., Wang, L. & Zhou, J. 2013. Optimization of microwave-assisted enzymatic extraction of polyphenols from waste peanut shells and evaluation of its antioxidant and antibacterial activities in vitro. *Food and Bioproducts Processing,* 91**,** 158-168. <https://doi.org/10.1016/j.fbp.2012.09.003>

Zhang, J., Liu, L., Jiang, Y., Faisal, S., Wei, L., Cao, C., Yan, W. & Wang, Q. 2019. Converting peanut protein biomass waste into “double green” meat substitutes using a high-moisture extrusion process: A multiscale method to explore a process for forming a meat-like fibrous structure. *Journal of agricultural and food chemistry,* 67**,** 10713-10725. <https://doi.org/10.1021/acs.jafc.9b02711>

Zhang, X., Lian, H., Shi, J., Meng, W. & Peng, Y. 2020. Plant extracts such as pine nut shell, peanut shell and jujube leaf improved the antioxidant ability and gas permeability of chitosan films. *International journal of biological macromolecules,* 148**,** 1242-1250. <https://doi.org/10.1016/j.ijbiomac.2019.11.108>

Zhang, Y., Wu, X., Huang, C., Zhang, Z. & Gao, W. 2022. Isolation and identification of pigments from oyster mushrooms with black, yellow and pink caps. *Food Chemistry,* 372**,** 131171. <https://doi.org/10.1016/j.foodchem.2021.131171>

Zhao, J., Zhou, T., Zhang, Y., Ni, Y. & Li, Q. 2015. Optimization of arachin extraction from defatted peanut (*Arachis hypogaea*) cakes and effects of ultra-high pressure (UHP) treatment on physiochemical properties of arachin. *Food and Bioproducts Processing,* 95**,** 38-46. <https://doi.org/10.1016/j.fbp.2015.03.009>

Zhao, X., Wu, S., Gong, G., Li, G. & Zhuang, L. 2017. TBHQ and peanut skin inhibit accumulation of PAHs and oxygenated PAHs in peanuts during frying. *Food Control,* 75**,** 99-107. <https://doi.org/10.1016/j.foodcont.2016.12.029>

Zhu, Y., Wang, Z. & Zhang, L. 2019. Optimization of lactic acid fermentation conditions for fermented tofu whey beverage with high-isoflavone aglycones. *LWT,* 111**,** 211-217. <https://doi.org/10.1016/j.lwt.2019.05.021>

Zuo, S.-S., Niu, D.-Z., Ning, T.-T., Zheng, M.-L., Jiang, D. & Xu, C.-C. 2018. Protein enrichment of sweet potato beverage residues mixed with peanut shells by *Aspergillus oryzae* and *Bacillus subtilis* using central composite design. *Waste and biomass valorization,* 9**,** 835-844. <https://doi.org/10.1007/s12649-017-9844-x>
